# Case Report: Diagnosis of leptospirosis presenting as aseptic meningitis using metagenomics CAPture sequencing

**DOI:** 10.3389/fmed.2026.1734396

**Published:** 2026-04-15

**Authors:** Shuang Wu, Mengling Wu, Wenhui Li, Chenrui Zhang, Ying Bi, Yuanteng Fan, Yan Xu, Dan He

**Affiliations:** 1 Department of Neurology, Zhongnan Hospital of Wuhan University, Wuhan, China; 2 Department of Rehabilitation Medicine, Wuhan Orthopaedics Hospital of Integrated Traditional Chinese and Western Medicine (The Affiliated Hospital of Wuhan Sports University), Wuhan, China

**Keywords:** leptospirosis, neuroleptospirosis, aseptic meningitis, MetaCAP, metagenomic next-generation sequencing, zoonotic infection

## Abstract

**Background:**

Leptospirosis is a globally prevalent zoonotic disease caused by pathogenic Leptospira species. The manifestation of leptospirosis can range widely, from being asymptomatic to causing severe multi-organ failure with a high mortality rate. It is uncommon for leptospirosis to present primarily with neurological complications. In this context, we discuss a notable case of *Leptospira borgpetersenii* infection manifesting as aseptic meningitis in China.

**Case presentation:**

In this study, we describe a primary case of neuroleptospirosis leading to symptomatic aseptic meningitis following exposure to *Leptospira borgpetersenii*. Initially managed for viral meningitis, the diagnosis of leptospirosis was subsequently confirmed through cerebrospinal fluid (CSF) analysis using metagenomic next-generation sequencing (mNGS) and Metagenomics CAPture Sequencing (MetaCAP), both of which identified *Leptospira borgpetersenii*. Following a course of antibiotics and methylprednisolone therapy, the patient fully recovered.

**Conclusion:**

This case underscores the importance of considering leptospirosis in differential diagnoses for aseptic meningitis, especially in individuals with occupational risks related to water or animal exposure. MetaCAP’s extensive coverage, sensitivity, and early pathogen detection capabilities can significantly enhance patient outcomes.

## Introduction

Leptospirosis, a prevalent global zoonotic disease, is caused by infection with pathogenic Leptospira species. Transmission to humans typically occurs through contact with water or soil contaminated by the urine of infected mammalian hosts, via abraded skin or mucosal surfaces (1). *Leptospira interrogans*, *Leptospira borgpetersenii*, and *Leptospira kirschneri* are the primary pathogens responsible for most human leptospirosis cases (2). Clinical manifestations of the disease range widely, from asymptomatic infections to severe multi-organ failure with a high mortality rate (3). Neurological complications occur in approximately 10–15% of cases, with aseptic meningitis being the most common (4, 5). The diagnosis was confirmed by a positive serological and/or etiological test with the Leptospirosis. Leptospirosis presents a protean clinical manifestation, ranging from asymptomatic to a severe disease requiring intensive care (6). Thus, leptospirosis was frequently underrecognized or misdiagnosed due to paradoxical clinical manifestations (7). With the enhancement of living standards and improvements in hygienic conditions, the incidence of Leptospirosis in clinical settings is becoming increasingly rare.

As a new approach, metagenomic next-generation sequencing (mNGS) is characterized by high sensitivity, rapid detection, and a reduced influence of prior antibiotics. mNGS is particularly useful for detecting rare, atypical, and complex pathogens (8, 9). Comparatively, targeted NGS (tNGS) has the advantage of simplicity and expediency (10). Metagenomics CAPture (MetaCAP) is an upgraded new generation of pathogen nucleic acid sequencing technology, which is based on the conventional mNGS and probe capture technology. In this report, we present a case of primary neuroleptospirosis resulting in symptomatic aseptic meningitis after exposure to *Leptospira borgpetersenii*. We used MetaCAP to identify a treatable, albeit rare, bacterial cause of aseptic meningitis. In this case, the results of next-generation sequencing directly affected the patient’s diagnosis and treatment in a significant way, resulting ultimately in a favorable outcome,

## Case presentation

A 49-year-old male patient was admitted to the hospital due to persistent headaches lasting for 2 days. The headache mainly manifests as a feeling of pressure throughout the head, accompanied by pain around both eyes, worsening at night. There were no symptoms of nausea or vomiting, no blurriness or double vision, no fever or chills, and no numbness or weakness in the limbs. Moreover, the patient was previously in good health, with a history of hypertension and diabetes, both of which were well-controlled with medication. Two weeks prior to this headache episode, the patient experienced a high fever accompanied by chills for 2 days, without receiving any specific treatment, and subsequently improved on their own. It is particularly noteworthy that the patient’s occupation involves selling aquatic products, which may lead to instances where an injured hand comes into contact with river or lake water.

Upon admission, we conducted a physical examination for the patient. Physical examination revealed: height 160 cm, weight 70 kg, blood pressure 129/76 mmHg, body temperature 36.6 °C, and clear consciousness (oriented with Glasgow Coma Scale 15/15). Pupils were equal, round, and reactive to light, with normal eye movements and no nystagmus observed. Facial symmetry was noted with equal bilateral frontal wrinkles and nasolabial folds. The strength and tone in all four limbs were normal. Both superficial and deep sensations were intact, with symmetric tendon reflexes in the limbs. Physiological reflexes were present, Babinski’s sign. The Kernig’s sign and Brudzinski’s sign were negative, although he had neck stiffness.

**Figure 1:**
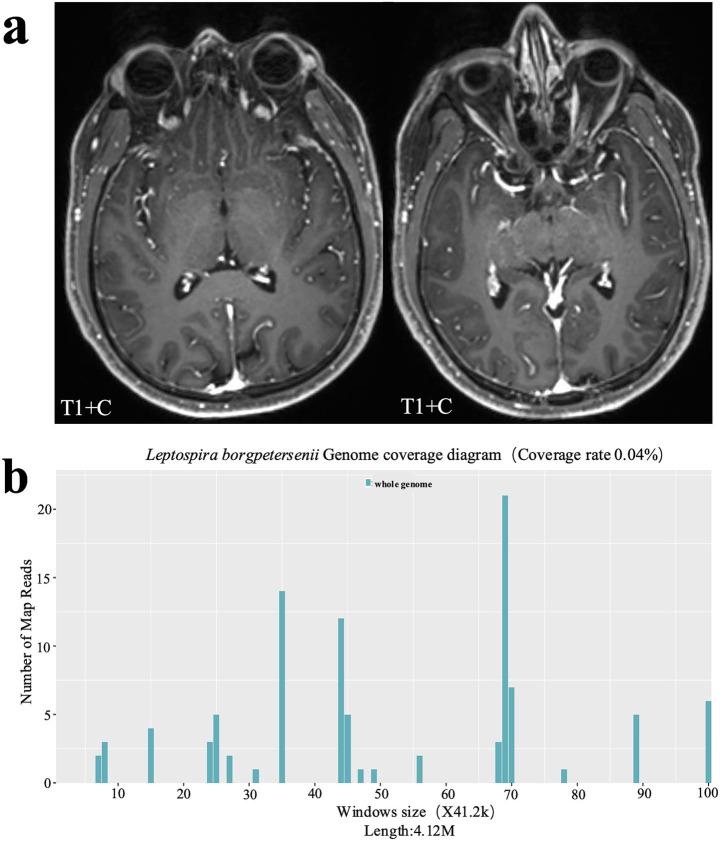
Imaging findings and sequencing result of the patient. **(a)** Magnetic resonance imaging (MRI) of the brain revealed mild dural and leptomeningeal enhancement. T1 + C, contrast-enhanced T1-weighted imaging. **(b)** MetaCAP indicated 34 sequence reads of *Leptospira borgpetersenii* were identified with a coverage rate of 0.04%, which supported the diagnosis of a *Leptospira borgpetersenii* infection manifesting as aseptic meningitis.

At admission, peripheral blood examination demonstrated white blood cell count was normal at 6.09 × 10^9^/L (normal range: 3.5–9.5 × 10^9^/L), but with a decrease in lymphocyte count (0.76 × 10^9^/L, normal range: 1.1–3.2 × 10^9^/L) and percentage (12.4%, normal range: 20–50%) whereas an increase in neutrophil percentage (76.2%, normal range: 40–75%). Tests of liver and kidney function were normal. Meanwhile, hypokalemia (potassium 3.29 mmol/L, normal range: 3.5–5.5 mmol/L), hyponatremia (sodium 136.4 mmol/L, normal range: 137–147 mmol/L), hypochloremia (serum chloride 97.6 mmol/L, normal range: 99–110 mmol/L) and hyperuricemia (uric acid 475.0 μmol/L, normal range: 208–428 μmol/L), with elevated creatinine kinase (244 U/L, normal range: <171 U/L), were detected. Infection-related markers showed that hypersensitive C-reactive protein was normal (2.59 mg/L, normal range: 0–3 mg/L). Screenings for infectious diseases, including hepatitis A virus core antigen, hepatitis B surface antigen, hepatitis C antibody, HIV antigen/antibody, and *Treponema pallidum* antibody, were negative. On investigating, his cerebrospinal fluid (CSF) analysis revealed an increase in CSF opening pressure, protein content, lactic acid and WBC count, as follows: 230 mmH_2_O (normal range: 80–180 mmH_2_O); CSF protein 0.80 g/L (normal range: 0.15–0.45 g/L); lactic acid 3.27 mmol/L (normal range: 1.0–2.8 mmol/L); white blood cell: 297 × 10^6^/L (normal range: 0–8 × 10^6^/L); CSF glucose was 3.21 mmol/L (normal range: 2.5–4.48 mmol/L). Cytology of CSF revealed 45% neutrophil, 37% lymphocyte and 18% monocytes. Screening for common pathogens in the CSF, including polymerase chain reaction (PCR) for herpes simplex virus (HSV), cytomegalovirus (CMV), and Epstein–Barr virus (EBV); bacterial and fungal cultures; and CSF staining (Gram stain, India ink stain for Cryptococcus, and acid-fast stain for bacteria), was entirely negative. In terms of magnetic resonance imaging (MRI) of the head, there were nonspecific mild dural and leptomeningeal enhancement (Figure 1A). B-scan ultrasonography revealed that the lymph nodes of the left axilla and bilateral neck were enlarged. Considering the low suspicion for bacterial meningitis, empirical antimicrobial treatments were not given to the patient.

Based upon the clinical and laboratory findings, the patient received 250 mg of ganciclovir q12h and 25 g of mannitol q8h for meningitis. However, 5 days later, his symptoms was not improved or even worsened after receiving the initial treatment. Specifically, the severity of headache remained unchanged, and intermittent low-grade fever was observed, with the highest recorded temperature reaching 37.8 °C. After obtaining written informed consent, the patient was included in the study for pathogen detection and discovery in hospitalized patients with the use of MetaCAP. Within 48 h after receipt of the clinical samples, Metagenomics CAPture sequencing analysis of million probe with the use of a bioinformatics pipeline for the detection of more than twenty thousand all known pathogenic microorganisms detected sequence reads corresponding to leptospira infection in the patient’s CSF but not in the serum. Therefore, the patient’s treatment regimen was modified 1 week after admission. The patient was managed to complete a 14-day course of intravenous ceftriaxone, as well as treatment with prednisolone (40 mg/day). The patient improved significantly after 2 days of treatment.

## Methods

**Figure 2:**
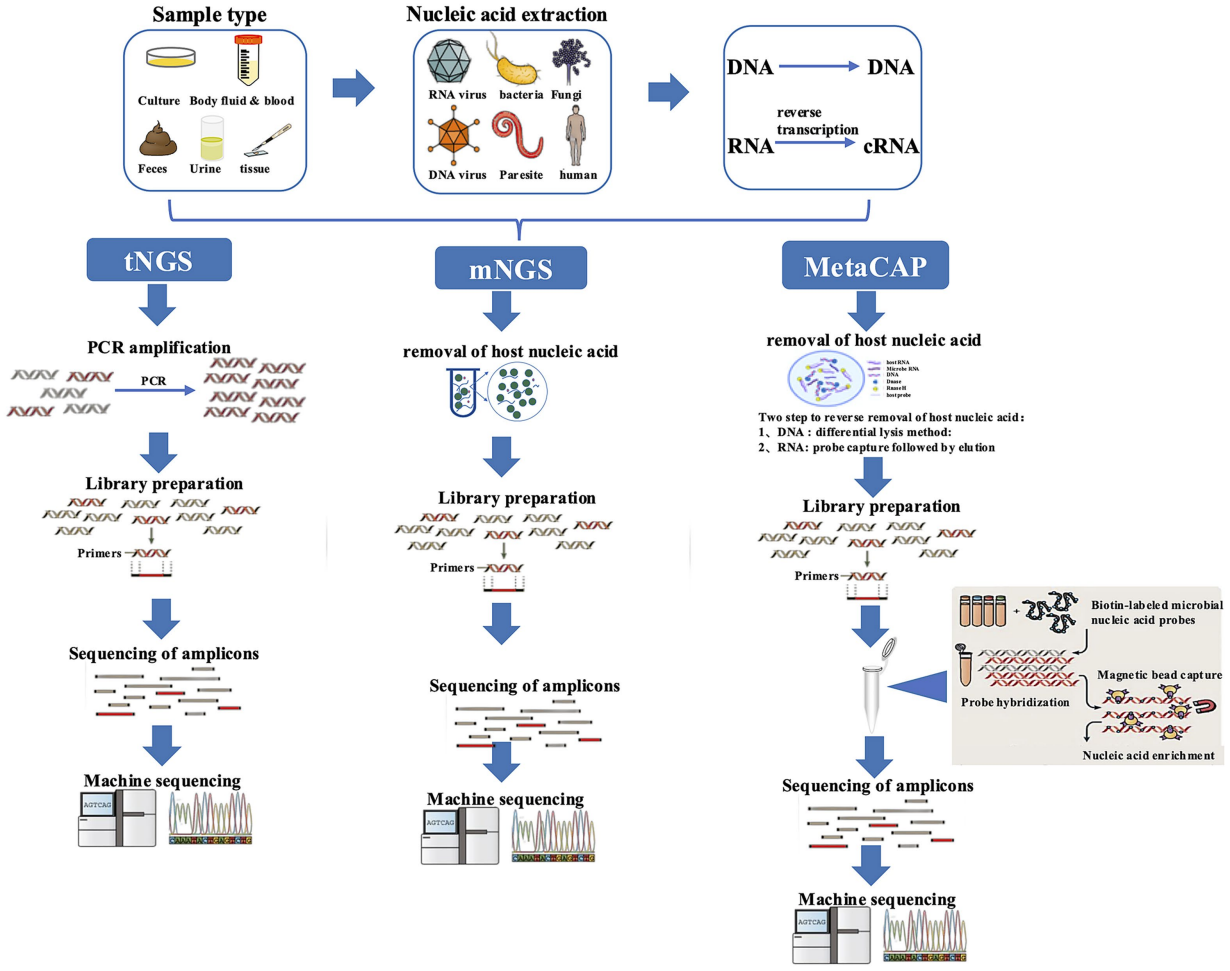
The flow chart of tNGS, mNGS, and Metacap (provided by Guangzhou Kingmed Center for Clinical Laboratory). mNGS, Metagenomic next-generation sequencing; tNGS, targeted next-generation sequencing; MetaCAP, Metagenomics CAPture; PCR, polymerase chain reaction.

The sample was sent to the Guangzhou Kingmed Center for Clinical Laboratory for analysis by MetaCAP. Metacap is capable of simultaneously detecting DNA and RNA. Amplified DNA and RNA libraries for MetaCAP sequencing were constructed from extracted nucleic acid derived from clinical samples as described previously, followed by library validation and sequencing on a mainstream sequencing platform. As part of the routine workflow, negative controls (non-template controls) and positive controls are processed in parallel with clinical samples in each sequencing batch to monitor for potential contamination during nucleic acid extraction, library preparation, and sequencing. Combined with differential technology to subtract human host sequences, pathogen sequences in samples were enriched in two-way enrichment. Bioinformatics analysis was used to remove human reads through aligning them to the human genome (computational host subtraction). Millions customized probes are used by Metacap to hybridize with microbial nucleic acids, for the comprehensive identification of more than 20,000 pathogens (including bacteria, viruses, fungi, and parasites) (Figure 2).

## Results

MetaCAP is a novel pathogen detection technology that based on the combination of probe capture and NGS. There are 14,330 bacteria, 814 fungi, 15,720 viral taxa, and 169 parasites in its classification reference databases. In the CSF of patient, MetaCAP indicated 34 sequence reads of *Leptospira borgpetersenii* were identified with a coverage rate of 0.04% (Figure 1B). What is more, its relative abundance was 98.38% with a confidence level of 99%. Even though there were only 34 reads, despite low gene coverage, based on the patient’s contaminated water exposure history, headache, stiff-neck and fever, *Leptospira borgpetersenii* was diagnosed in time. Meanwhile, we employed the enzyme-linked immunosorbent assay (ELISA) to quantify Leptospira-specific IgM antibodies, and the results demonstrated a positive reaction(+). We diagnosed the patient with leptospirosis meningitis finally. One month after discharge, we conducted a follow-up and found that the patient had no discomfort or sequelae. The patient was followed-up for 3months after discharge, and there was no discomfort or sequelae of treatment.

## Discussion

As a zoonotic disease, Leptospirosis exhibits a global prevalence and diverse clinical presentations (11). Pathogenic leptospires can live in a variety of animal hosts in nature, which causes Leptospirosis (12). Typically, humans serve as the secondary host and acquire infection through contact with contaminated water, urine, blood, or tissue originating from infected rodents (13). Among the pathogenic Leptospira species, there are more than 350 serovars and 30 pathogenic and saprophytic serotypes (14). Leptospirosis exhibits a wide distribution and demonstrates a higher incidence in tropical countries primarily attributed to occupational exposure where it is more commonly associated with recreational activities such as water rafting and swimming (14, 15). The organisms have the ability to infiltrate compromised skin or intact mucous membranes, subsequently entering the circulatory system and rapidly spreading to diverse tissues (16). Considering the occupation of our patient, the source of infection is most likely attributable to personal exposure to river water that has been contaminated. However, with improvements of living standards and changes in lifestyle, the incidences of leptospirosis decreased. In this report, we present typical a case of primary neuroleptospirosis resulting in symptomatic aseptic meningitis after exposure to *Leptospira borgpetersenii*. Thus, this case suggests that physicians should consider leptospirosis as a possible diagnosis when there are routes of infection.

As a biphasic disease, the incubation periods of Leptospirosis can range from 2 to 30 days with an average incubation period of 5–14 days. The disease is characterized by an initial leptospiraemic phase lasting for a period of 3–7 days, which is subsequently followed by an immune phase spanning from 4 to 30 days. The first phase (lasts for a week), known as the leptospiremic or septicemic phase, exhibits an abrupt onset, symptoms include fever, headaches, chills, skin rashes, conjunctival suffusion and myalgia. During the second phase (last up to 30 days), known as the immune phase, leptospirosuria occurs and anti-Leptospira antibodies are produced. The patient experiences a relapse, characterized by the recurrence of fever and other constitutional symptoms. Furthermore, there is a possibility of the pulmonary, renal, cardiovascular, and neurological systems being affected (17). The CSF during this phase is characterized by normal glucose levels and protein levels of 50–100 mg/dL (18). In this case, the distribution of 45% neutrophil, 37% lymphocyte and 18% monocytes in CSF particularly attracted our attention. Meanwhile, the empirical antiviral therapy was not efficient. These results prompted us to undertake a CSF next-generation sequencing technology and eventually tested positive for *leptospira borgpetersenii*.

Neurological manifestations associated with leptospirosis encompass encephalitis (altered sensorium, seizures, psychosis, hemiplegia), intracranial bleeds, cerebellitis, movement disorders, myelitis, flaccid paraplegias (like Guillain-Barré syndrome), neuralgias, autonomic lability, mononeuritis, polymyositis, and acute disseminated encephalomyelitis (4, 19). As previously mentioned, approximately 80% of patients have aseptic meningitis with or without symptoms during the immune phase of the illness. It is rare to present severe neurologic complications such as coma, meningoencephalitis, hemiplegia, transverse myelitis or Guillain-Barré syndrome (18). Although 80% of cases of leptospirosis have meningeal signs (4), this case is a rare instance of manifests primarily as a neurological disorder. Our patient, who had risk factors associated with exposure to river water that has been contaminated and his occupation as fish monger, presented with aseptic meningitis with normal liver and kidney function. We used the modified Faine’s criteria to evaluate the patient, which may help detection of leptospirosis. Modified Faine’s criteria consists of three parts: part A (the clinical history), part B (epidemiological history) and part C (supported by laboratory parameters). If part A or the total of parts A and B is/are higher than 26, a presumptive diagnosis can be made. Leptospirosis can also be suspected when a total score of parts A, B, and C exceeds 25 (20). The calculated score of the patient was 29, which is higher than 25.

Recently, metagenomic next-generation sequencing (mNGS) has been reported to be successful in diagnosing leptospirosis, which provides a good reference for solving this problem (21). However, it is still noteworthy that mNGS would bring confusion in report interpretation while the detection rate of pathogens was improved significantly. The identification of the pathogenic bacteria would be difficult when multiple pathogen sequences are detected simultaneously. What is more, there are some limitations, such as the choice about dual-process selection of DNA and RNA sequencing, the low sensitivity caused by a high proportion of human nucleic acid, false negatives for resistance virulence (22) and expensive testing costs. Targeted next-generation sequencing (tNGS) uses multiplex PCR for target enrichment of pathogens, it achieves simultaneous detection of DNA and RNA. It can decrease the amplification of unwanted human sequences and enable precise diagnosis. However, due to primer cross-interference, its pathogen spectrum range is limited (no more than 1,000 types of pathogens) (23). MetaCAP is a next-generation pathogen nucleic acid sequencing product that is comprehensively upgraded based on conventional mNGS through probe capture technology. With its large number of probes, it can simultaneously detect more than 20,000 pathogens at once. MetaCAP is characterized by its broad spectrum coverage, high sensitivity, ability to identify viral homologous mutations, coverage of drug-resistant sites in some special pathogens, and combines the benefits of both mNGS and tNGS. Likewise, owing to the nucleic acids of the samples may be randomly fragmented, MetaCAP may have poor specificity and even false positive results caused by the potential hybridization with fragments containing partial target regions. In addition, although the MetaCAP has obvious advantages, if encountering completely unknown pathogens, mNGS is the better choice (24). In conclusion, it is imperative for clinicians to carefully select the appropriate detection method that aligns with the specific clinical scenario in order to enhance the accuracy of diagnosis and treatment for patients.

## Data Availability

The raw data supporting the conclusions of this article are not publicly available due to patient privacy restrictions. Data may be made available by the corresponding author upon reasonable request.
